# Explant of a Chronic Atlantoaxial Implant Infection in a Dog

**DOI:** 10.1155/2023/1942540

**Published:** 2023-07-11

**Authors:** Thao Vo, Gabriel Garcia, Sheila Carrera-Justiz

**Affiliations:** Small Animal Clinical Sciences, University of Florida, College of Veterinary Medicine, Gainesville, FL 32610, USA

## Abstract

An 11-year-old male neutered Yorkshire Terrier was presented with a cervical mass that developed a draining tract. Aside from the dysphagia reported by the owner, his neurologic exam was normal. Three years prior, the patient was diagnosed with an atlantoaxial subluxation that was ventrally stabilized with polymethylmethacrylate (PMMA) and self-tapping titanium screws. There were no postoperative complications until presenting with the cervical mass and dysphagia. Computerized tomography (CT) of the cervical spine confirmed caudal migration of the PMMA and screws with an abscess surrounding the implant. A surgical explant of the PMMA and screws was performed without complication. The atlantoaxial joint remained normally aligned on postoperative radiographs. Cultures of the implant grew *Streptococcus bovis*. He was treated with cephalexin (22 mg/kg PO BID) for 30 days. At the time of his one-month recheck, he was swallowing normally with no neurologic deficits. He remains normal at the time of this report (17 months later). This case reports a successful explant of a chronic atlantoaxial implant infection.

## 1. Introduction

Atlantoaxial instability (AAI) is a well-recognized problem in primarily young, toy-breed dogs in veterinary medicine [1, 2]. Common causes of AAI often involve malformation of the ligaments and bones involved in articulating the first and second cervical vertebrae with or without a history of trauma [1, 2]. Clinical signs range from cervical pain to tetraparesis, ataxia, and vestibulocerebellar signs [1]. Evidence of instability may be readily evident in lateral cervical radiographs. Alternatively, a CT may be indicated in cases with a history of trauma and for surgical planning.

Although medical management is an option for treatment of AAI, various surgical techniques have been developed over the past several years to find the ideal approach to reduce postoperative complications [3–7]. Ventral stabilization with screws and polymethylmethacrylate and immediate postoperative complications have been described [3–5, 7]. However, literature on AA implant infections is lacking in the immediate postoperative period and chronically. The objective of this paper was to describe a chronic implant infection in a dog with successful removal of the implant and treatment of the infection.

## 2. Case Presentation

An 8-year-old male neutered Yorkshire Terrier presented to the University of Florida in May 2018 for an acute onset of cervical pain and ataxia. On neurologic exam, the dog was ambulatory with tetraparesis and cerebellovestibular ataxia. There were delayed to absent postural reactions on all four limbs in the absence of cervical pain on palpation (full range of motion not assessed). Neuroanatomic localization was to the cranial cervical spine. Beside historical tracheal collapse and an oronasal fistula, there were no significant medical comorbidities. Preliminary diagnostic tests (complete blood cell count, chemistry, and thoracic radiographs) were unremarkable.

**Figure 1:**
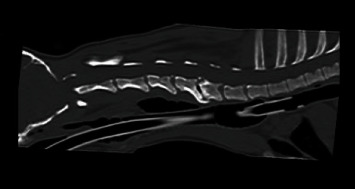
Computerized tomographic image of the cervical spine on the midsagittal plane. Note the dorsally displaced axis and the sclerotic end plates at the C5-C6 interface.

A computerized tomographic (CT) (Toshiba Aquilion, 160-slice helical CT) study of the cervical spine revealed malalignment of the atlantoaxial joint with dorsal displacement of C2 and widening of the space between C1 and C2. The dens were subjectively small in size. Additionally, the C5-6 intervertebral disc space was narrowed and showed a vacuum phenomenon. The adjacent endplates were sclerotic and mildly irregular (Figure 1). Based on these findings, he was diagnosed with atlantoaxial subluxation and suspected diskospondylitis at the C5-6 interspace. Urine culture revealed no growth. A cervical splint was placed, and he was started on a tapering anti-inflammatory dose of prednisolone (0.5 mg/kg/d tapered over two weeks) and cephalexin (27 mg/kg twice daily) prior to surgery.

**Figure 2:**
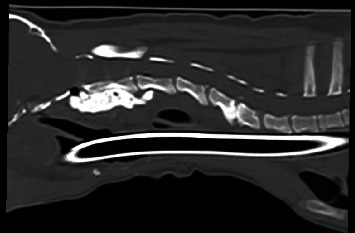
Immediate postoperative CT of the cervical spine on the sagittal plane.

Surgical stabilization was achieved with ventral placement of screws (self-drilling and self-tapping titanium screws) (CMF Medicon, Jacksonville, Florida) and polymethylmethacrylate (PMMA) (Teknivet, France). A postoperative CT confirmed appropriate reduction of the atlantoaxial joint and placement of screws with PMMA (Figure 2). One day postoperatively, the patient was noted to have Horner's syndrome OD, and the remainder of his neurologic exam was static. The patient was discharged two days postoperatively on gabapentin (14 mg/kg three times daily for two weeks) and cephalexin (27 mg/kg twice daily for one month).

**Figure 3:**
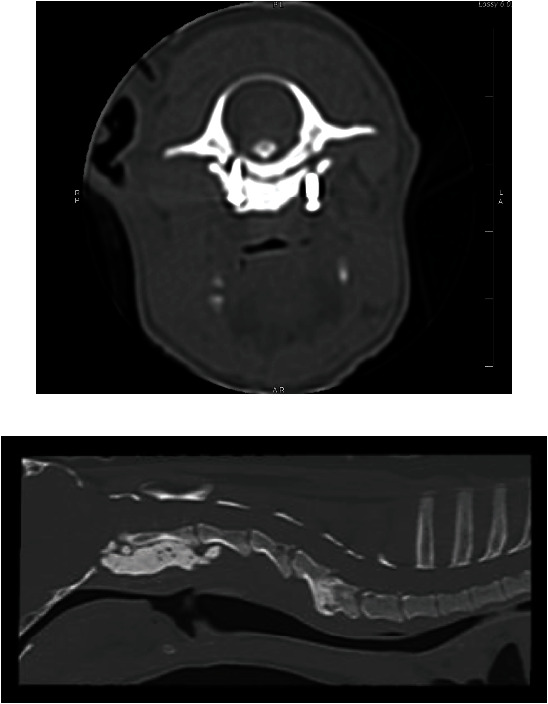
Recheck CT six months postoperatively. (a) Note the ventral displacement of the cranial-most screw on the left aspect of the body of C1. (b) Note the progressive collapse of the C5-6 disc space.

The dog presented for a recheck CT six months after surgery. The CT revealed that the cranial-most screw on the left aspect of the body of C1 was ventrally displaced about 4 mm. The atlantoaxial joint remained normally aligned, and the remainder of the screws and PMMA were unchanged. There was progressive collapse of the C5-6 disc space and ventral displacement of the cranial end plate and dorsal lamina of C6 relative to C5 (Figure 3). Clinically, the dog was neurologically improved with only mild tetraparesis and ataxia noted at that time.

**Figure 4:**
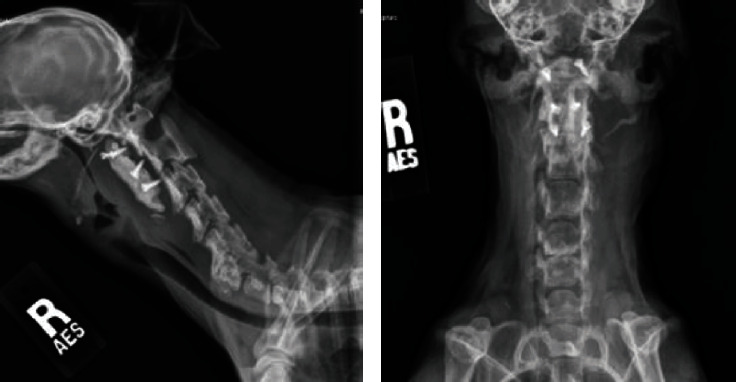
Digital radiographs of the cervical spine in lateral (a) and dorsal (b) views. Note the caudoventrally displaced implant.

The dog presented to the emergency service three years later after the owners noted a new mass at the caudoventral aspect of his left ear, adjacent to the wing of C1. His neurologic examination was normal. Cervical radiographs revealed caudoventral migration of the implant and regional left-sided soft tissue swelling (Figure 4). The atlantoaxial joint remained anatomically aligned. The cytology of the mass revealed neutrophilic inflammation with no infectious organisms observed. The emergency clinician gave the recommendation to monitor the mass at home at that time.

**Figure 5:**
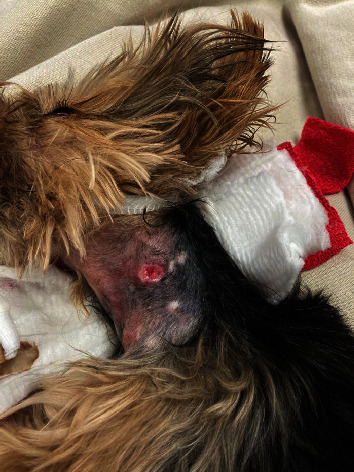
Open draining tract on the left lateral aspect of the neck.

**Figure 6:**
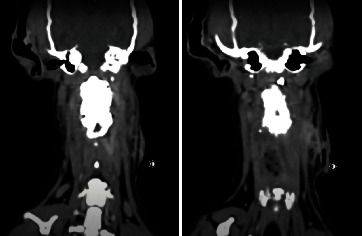
CT of the cervical spine, dorsal view. Note the draining tract on the left lateral aspect of the implant.

Two weeks later, the dog was reexamined for the development of a draining tract associated with the cervical mass and dysphagia (Figure 5). Cervical radiographs revealed a gas tract surrounding the left lateral aspect of the implant. There was moderate thickening of retropharyngeal soft tissue adjacent to the implant, causing ventral deviation and compression of the laryngopharynx and larynx. The cytology of the mass revealed degenerative neutrophils and rare intracellular cocci doublets. The culture of this draining tract wound grew scant *Staphylococcus epidermidis*. The neurologic examination was normal, and the dog was transferred to the neurology service for a cervical CT scan which confirmed that the PMMA and screws had migrated ventrally and were no longer in contact with C1-C3 (Figure 6). There was an abscess surrounding the migrated implant with an associated draining tract. This abscess was characterized by a well-defined, fluid-attenuating structure with a thick, contrast-enhancing rim which extended cranially, adjacent to the ventral aspect of the occipital condyles, and caudally to the level of C4. Clindamycin (14 mg/kg orally twice daily) treatment was prescribed for the next 7 days, and the owners were advised to return in one week for an explant.

**Figure 7:**
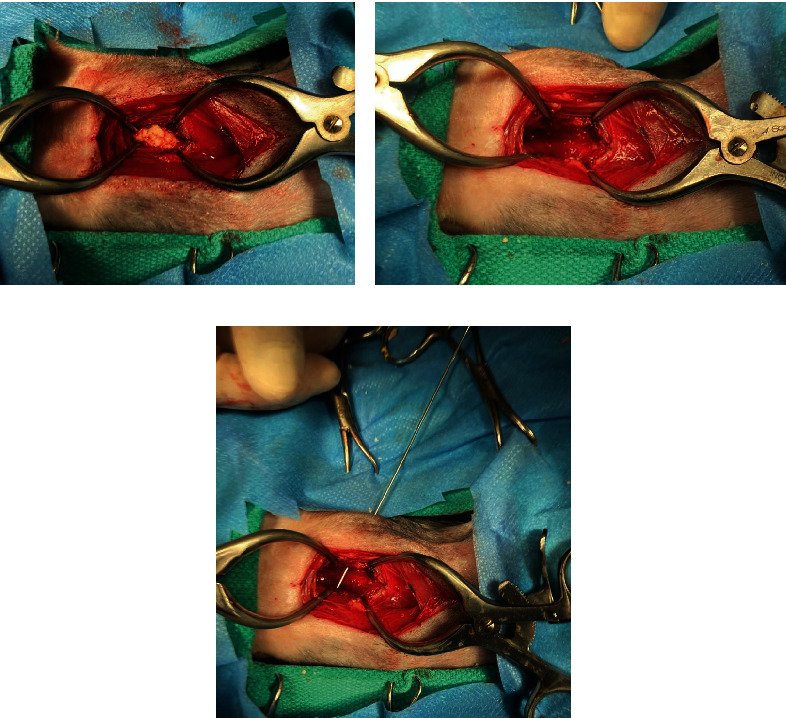
(a) Upon clearing the surrounding fibrous capsule, the implant was identified. (b) Removal of the explant. (c) Pin demonstrating the communication between the external wound and draining tract to the region of the implant.

On physical examination one week later, the draining tract was closed, and the neurologic examination remained normal. The atlantoaxial space was approached, and the longus colli and fibrous tissue were carefully dissected to reveal the implant. There was dark hemorrhage and serosanguineous fluid associated with the implant upon initial incision of the fibrous capsule. Gelpi retractors were placed at the cranial and caudal aspects of the implant (Figure 7). Additional fibrous tissue at the cranial aspect of the implant was dissected/incised using a #15 blade. Lempert rongeurs were used to break down the caudal aspect of the PMMA surrounding the 2 most caudal screws. The remainder of the implant was removed in one piece with gentle traction with Lempert rongeurs. Samples for culture were taken of the implant and the fluid surrounding it. The implant was submitted for culture as well. The area was flushed with sterile saline.

**Figure 8:**
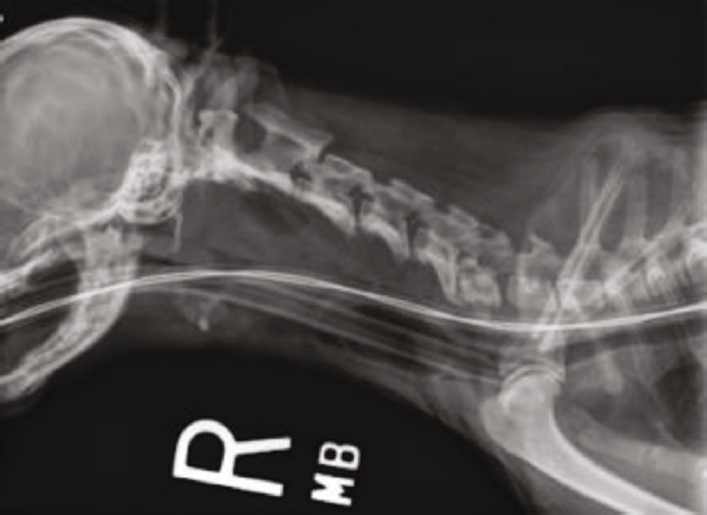
Lateral cervical radiograph immediately postoperative explant. Note the normal alignment between C1-C2 is maintained.

A butterfly catheter was fenestrated and placed in the dead space from the implant removal. The drain was anchored with a Chinese finger trap. Postoperative radiographs were performed, and there was no evidence of malalignment (Figure 8). Recovery was uneventful, and the dog remained neurologically normal.

Aerobic culture of the implant showed scant growth of *Streptococcus bovis*. He was started on cephalexin (22 mg/kg orally twice daily) in addition to the clindamycin for the next 30 days. The dog presented for a recheck examination one month later and was found to be comfortable and neurologically normal. At the time of this report (17 months after surgery), the patient continues to do well.

## 3. Discussion

This case highlights the clinical, diagnostic, and surgical findings in a case of chronic atlantoaxial implant infection. The chronicity of this infection (three years after atlantoaxial stabilization) has not been previously reported in veterinary medicine, to the best of the authors' knowledge. Long-term surgical complications described include implant migration, pin loosening and/or breakage, dyspnea, and dysphagia [3–5, 8]. These complications presented over a span of one month to 11 months after surgery for stabilization of the atlantoaxial joint.

One reported dog developed a ventral cervical draining tract and became nonambulatory tetraparetic 11 months after surgical stabilization with positively threaded profile pins and PMMA [4]. This same dog's radiographs showed no evidence of pin breakage or implant migration. Two cultures of the draining tract showed no bacterial growth. After not showing any response to empiric antibiotics, the patient was started on a two-week course of anti-inflammatory prednisone, and the draining tract resolved. In another study [3], a dog developed a draining tract six months after surgery. CT confirmed caudal migration of the caudal C2 screw with maintenance of the osseous fusion of C1-C2. The implant was removed, and the patient was normal at the time of the recheck. In the same study, another dog developed cervical pain. Radiographs revealed a broken transarticular pin and migration of another pin. These pins were removed, and the patient was normal seven months later. There was no description of cultures or antimicrobial treatments for these patients.

More chronic complications have been described with implants in the thoracolumbar spine. Commonly reported minor complications include partial screw loosening or breakage without worsening of the patient's neurologic status or evidence of implant failure [9, 10]. Major complications described included pain and swelling in the region of the implant with or without exposure of a part of the implant. In one patient undergoing spinal segmental stabilization for congenital vertebral malformation, there was a swelling noted over the implant two years postoperatively that resolved with nonsteroidal anti-inflammatory medication and rest. In this same dog, the swelling reoccurred three years later. Radiographs revealed focal lysis of the spinous processes adjacent to the implant. The implant was surgically removed and the culture obtained, but the results were not reported in this paper [11]. In another case series assessing the long-term outcomes of PMMA and pins/screw for spinal stabilization, exposure of the PMMA and/or development of a draining tract was noted in five patients over a span of six months to 1.5 years postoperatively [12]. Four of the five patients had surgery to remove the implant, and three of the cultures grew *E. coli*: coagulase-positive *Staphylococcus,* beta-lactamase *Staphylococcus* and methicillin-resistant *S. aureus* (MRSA). One patient had no growth. It is possible that there are more described instances of implant exposure and infection with thoracolumbar implants due to less soft tissue coverage of the implant (muscle dissection, epaxial muscle atrophy) and increased mobility of the spine at this point compared to the atlantoaxial joint. In addition, most causes of thoracolumbar injury may involve an exterior wound secondary to trauma (hit by a car or attacked by a dog) that could act as the source of infection.

In humans that required revisional craniocervical junction surgeries, 13 of 55 (23.6%) patients required revisional surgery to remove an infected implant [13]. The use of sublaminal wires, the inclusion of the occiput in the fusion, and transoral decompression preceding posterior fixation were significantly associated with the development of an implant infection requiring surgical removal [13]. The duration between the first surgery and the revisional surgery was variable and ranged from one year to 19 years. In general, implant migration or infection was not noted until at least one year after surgery in humans. Although the time frame for the occurrence of implant migration and/or infection is also variable in veterinary medicine, the earliest time frame reported was about six months postoperatively. The most prolonged time period is described in this paper. It is possible that these differences are due to the surgical approach (inclusion of the occiput, transoral approach, types of implants used) and the shorter life span of dogs.

The culture obtained from the implant grew *Streptococcus bovis.* This is different from the initial organism (*Staphylococcus epidermidis)* isolated when the draining tract was cultured. This is suspected to be due to the swab obtaining a superficial bacteria sample. In a study comparing cultures obtained from swabs and biopsies of wounds, the culture results were identical in 21.2% of the cases [14]. *S. bovis* is a commensal bacterium found in the gastrointestinal tract of humans and ruminants, among other animals [15]. In humans, *S. bovis* has been a notable cause of bacteremia and endocarditis. *S. bovis* has rarely been isolated in urinary tract infections and mitral valve endocarditis in dogs and cats [15, 16]. One of those dogs with mitral valve endocarditis was noted to have concurrent sternebral osteomyelitis [15]. In another study, a dog acquired infective endocarditis (*S. bovis)* immediately following a dental procedure [16]. Although this is unlikely with the dog in the current study due to the absence of teeth, other possible sources to consider would be the presence of an oronasal fistula and suspect diskospondylitis. However, the oronasal fistula was present since birth, and the diskospondylitis for several years. The patient in this study had a negative urine culture, though an echocardiogram and blood cultures were not performed. The source of *S. bovis* infection in this dog remains unknown.

Despite the presence of an implant infection, the patient remained neurologically normal, with the only abnormal findings being mild cervical pain, the palpable cervical mass/abscess, and draining tract formation. This is similar to dogs in previous studies that represented mostly because of the owners' ability to palpate and/or visualize a portion of the implant under the skin or the presence of a draining tract. Besides the one patient that became nonambulatory tetraparetic, the other dogs remained neurologically normal or had mild cervical pain. This is likely due to persistent C1-C2 stabilization, and the infection primarily involving the migrated parts of the implant.

## 4. Conclusion

In summary, atlantoaxial implant infections can occur several years postoperatively and should be considered a differential if there are physical and neurologic examination findings consistent with an abscess. Despite the presence of an implant-associated infection, patients rarely demonstrate neurologic deficits. The source of chronic infections can be challenging to determine, and obtaining a culture and sensitivity will help guide treatment with the appropriate antimicrobials. Removal of the implant is recommended to remove the nidus of infection that may be difficult for oral antimicrobials to penetrate.

## Data Availability

The data used to support the findings of this study were included in the article.
